# Quantitative Cross-Sectional Study About the Prevalence of Depression Among Epileptic Patients in Saudi Arabia

**DOI:** 10.7759/cureus.45491

**Published:** 2023-09-18

**Authors:** Reema Saleh A Albalawi, Sarah Mutlq O Alanzi, Amjad Fiusal H Alharthe, Sarah Hadi S Atawi, Rahaf Masoud D Albalawi, Hind Abdulaziz S Alanazi, Maram Saleh A Alsayed, Mohammad Zubair

**Affiliations:** 1 Medicine, University of Tabuk, Tabuk, SAU; 2 Medical Microbiology, University of Tabuk, Tabuk, SAU

**Keywords:** saudi arabia, prevalence, cross-sectional, epilepsy, depression

## Abstract

Background: There are high prevalence of mental health co-morbidities in people with epilepsy, with major depressive disorder being the most common among them.

Objective: This study aims to investigate the prevalence of depression among epileptic patients in Saudi Arabia. We also explored some of the sociodemographic and clinical variables associated with depression in epilepsy.

Methods: This was a cross-sectional analysis executed across the four geographical regions of Saudi Arabia and the sample size is calculated to be 358. Data collection was facilitated through a digital self-administered questionnaire, which consisted of three parts: patients' sociodemographic variables, clinical variables, and patient health questionnaire (PHQ-9) depression score. Data processing and analytical procedures are undertaken using the SPSS software.

Results: Of the participants, 311 responded: 65.6% were females, and 34.4% were males. Approximately 50.5% had a confirmed epilepsy diagnosis and were included in the PHQ-9 depression score analysis. Notably, 84.7% manifested depressive symptoms, with the severe category being predominant at 84.7% and moderate at 3.8%. An intriguing observation was the heightened prevalence among the younger demographic (16 to 24 years), registering at 34.4%, a figure nearly 3% superior to older age brackets.

Conclusion: The majority of participants manifested depressive symptoms, with a significant association noted between medication quantity and depression prevalence in epilepsy. It is imperative to broaden the scope of research, encompassing varied methodologies and spanning multiple urban centers, to procure more robust and generalizable conclusions.

## Introduction

Epilepsy is one of the most common neurological diseases affecting 65 million people. It is characterized by recurrent seizures, which are excessive electrical activity in the brain. This abnormal electrical activity results in episodes of involuntary movements that may be localized to one part of the body (partial) or involve the entire body (generalized). Loss of consciousness as well as bladder and bowel control sometimes occur [1]. The prevalence of the disease in Saudi Arabia is 6.54 per 1000 [2]. There is a high prevalence of mental health co-morbidities in people with epilepsy; major depressive disorder was the most common mood disorder with a prevalence of 24.2% [3]. 

Many worldwide epidemiological studies have found the prevalence of depression and anxiety higher among people with epilepsy. A meta-analysis study was conducted in 2018 to investigate the prevalence of depression in people with epilepsy in all Asian countries. Data were collected using "depression," "Epilepsy," and "country name" in the PubMed database for all Asian countries since 1947. The study included 687 studies, and the results showed a high prevalence of depression in adults with epilepsy [4]. Likewise in Africa and Europe, several studies done in Ethiopia, Guinea, Nigeria, and Scotland demonstrated similar findings [5-8]. Consequentially, a study done in 2012 found that suicide ideation is common among epilepsy outpatients; 10.9% had recent suicidal thoughts, and an additional 1.0% claimed they would have killed themselves if given the opportunity. In this study, depression was revealed to be a more reliable predictor of suicidal thoughts than seizure triggers or self-perceived quality of life [9].

Locally, a cross-sectional study was conducted in Taif City, Saudi Arabia from September 2020 to March 2021 to assess the prevalence of depression among people with epilepsy. The prevalence of depression was found to be 76.7%; a severe form of depression was identified in 8.7% and was more in people aged 25-34 years old [10]. This study included only one city; therefore, in order to obtain a more representative view, it must be done across multiple cities in Saudi Arabia. Our objective is to study the prevalence of depression among epileptic patients in Saudi Arabia. We also explore some of the sociodemographic and clinical variables associated with depression in epilepsy.

## Materials and methods

Study sampling and setting 

This was a cross-sectional study undertaken from January to May 2023 in Saudi Arabia. Data collection was facilitated using a self-administered electronic questionnaire. Prior to its commencement, the study obtained the requisite ethical approval from the Research Ethics Committee (REC) at the University of Tabuk. The primary objective was to determine the prevalence rate of depression in individuals with epilepsy within Saudi Arabia. Those who are 16 years of age or older with a diagnosis of epilepsy and provided consent were included. Individuals who declined participation or those who were incapable of providing informed consent were not included in the study. The sample size was determined using Kish's L. 1965 formula to be approximately 358 participants.

Kish's L. 1965 formula: \[ n = \left(Z_{1-\alpha}\right)^2 \cdot \frac{{P \cdot (1 - P)}^2}{{D^2}} \]

Where P, prevalence (due to the absence of data in the country, the proportion of epileptic patients living with depression was assumed to be 50%),=0.5; D2, precision,=5%=0.05; n, sample size, Z1-α; Z1-0.05=1.96 (from the table based on 95% confidence level).

Data collection

Data collectors were assigned from four different regions (North, South, East, and West) to obtain more representative information. They circulated the questionnaire over neurology clinics, pharmacies, and social media platforms. The self-administered electronic questionnaire consisted of two parts; the first part is patient sociodemographic data (age, gender, region, marital status, employment, education level, previous history of mental illness, co-morbidities, and governmental support) and clinical information about the seizure (duration, number of episodes in the last 6 months, type of epilepsy, number of medications used for epilepsy). The second part contains the Patient Health Questionnaire-9 (PHQ-9) score for depression, which includes nine questions and is divided into four sub-scores (None-mild-moderate-moderately severe-severe). Validated for the Arabic-speaking population [11].

Statistical analysis

The data underwent analysis through IBM's statistical package for social science (SPSS), specifically version 28 designed for Windows (IBM Corp., Armonk, NY). In the process of comparing variables, the relationship between categorical variables was determined using Pearson's chi-square test. A p-value of <0.05 is considered significant. 

## Results

This study sought to assess the prevalence of depression among epileptic patients in Saudi Arabia, focusing on the relationship between sociodemographic characteristics and depression prevalence. Our research encompassed 311 participants, with a diverse range of regions and demographics in the country. The sociodemographic details of our participants are captured in Table 1. Notably, the majority of participants were of Saudi nationality, constituting 95.5% of the study population. Furthermore, the largest representation came from the Central region (35.7%), followed by the West (22.2%) and South (18.0%). The study was heavily influenced by urban residents, with 87.8% of the participants living in urban areas. Gender distribution showcased a more substantial representation of females at 65.6%, while males made up 34.4% of the study population. Age-wise, the younger age groups of 16-24 and 25-34 had the highest representation, constituting 34.1% and 31.2% of the participants, respectively. In terms of marital status, single individuals formed the majority at 49.8%, closely followed by the married category at 45.0%. Regarding education, a significant 66.2% had attained a college education or above, which may provide insights into the literacy and awareness levels among the participants. Finally, concerning employment status, 46.0% of the individuals were gainfully employed, while students represented 28.9% of the study group.

**Table 1:** Comprehensive sociodemographic indicators of the respondents

Variable	Category	N	Percentage (%)
Nationality	Saudi	297	95.5
	Non-Saudi	14	4.5
	Total	311	100.0
Region	South	56	18.0
	East	30	9.6
	North	45	14.5
	West	69	22.2
	Central	111	35.7
	Total	311	100.0
Residence	Rural	38	12.2
	Urban	273	87.8
	Total	311	100.0
Gender	Female	204	65.6
	Male	107	34.4
	Total	311	100.0
Age Group	16-24	106	34.1
	25-34	97	31.2

The study explored various clinical aspects as shown in Table 2. Epilepsy was identified in 50.5% of the cases (n=157), with 49.5% (n=154) not having the condition. A distinction was made regarding mental illness history, with 64.6% (n=201) negative and 35.4% (n=110) positive. In terms of co-morbidities, 29.3% (n=91) had other health issues, while 70.7% (n=220) did not. Government support was received by 23.2% (n=72) of participants, not received by 74.6% (n=232), and information was not available for 2.3% (n=7). The duration of epilepsy was stratified into less than two years (45.3%, n=141), two to five years (17.7%, n=55), and more than five years (37.0%, n=115). With respect to seizures in the last six months, 66.9% (n=208) reported no occurrence, while 33.1% (n=103) experienced seizures. Further differentiation was made in terms of convulsion types, with 66.6% (n=207) of individuals uncertain, 21.5% (n=67) having generalized convulsions, and 11.9% (n=37) experiencing focal ones. The use of anti-epileptic medications was noted among 48.2% (n=150), with 51.8% (n=161) being abstaining. Finally, the categorization of participants based on the number of medications revealed three groups: no medication (37.9%, n=118), polytherapy (29.3%, n=91), and monotherapy (32.8%, n=102).

**Table 2:** Clinical characteristics of the participants

		N	%
Presence of epilepsy	No	154	49.5
	Yes	157	50.5
	Total	311	100.0
History of mental illness	No	201	64.6
	Yes	110	35.4
	Total	311	100.0
Co-morbidities	No	220	70.7
	Yes	91	29.3
	Total	311	100.0
Presence of government support	Missing	7	2.3
	No	232	74.6
	Yes	72	23.2
	Total	311	100.0
Duration of epilepsy	2-5 Years	55	17.7
	Less than 2 years	141	45.3
	More than 5 years	115	37.0
	Total	311	100.0
Incidence of seizures in the last six months	No	208	66.9
	Yes	103	33.1
	Total	311	100.0
Type of convulsions	Focal	37	11.9
	Don’t know	207	66.6
	Generalized	67	21.5
	Total	311	100.0
Use of anti-epileptic medications	No	161	51.8
	Yes	150	48.2
	Total	311	100.0
Number of medications	No medication	118	37.9
	Polytherapy	91	29.3
	Monotherapy	102	32.8
	Total	311	100.0

Within the study, the assessment of depression scores using PHQ9 results is revealed in Table 3. Approximately 84.7% (n=133) of the patients exhibited signs of depression. A severe form of depression was identified in 3.8% (n=6) of the patients, and a moderately severe form was observed in 17.2% (n=27) of the patients. Depression was analyzed across various levels of severity, categorized by age, gender, nationality, residence, duration of epilepsy, and type of epilepsy. Age revealed increasing prevalence from 16-24 to 25-34, with a decline in the 35-44 and 45 or above categories. Gender differences were marked by higher rates of mild depression in males (19.7%) compared to females (14.6%). Nationality illustrated a pronounced prevalence among Saudis (31.8%, mild), while non-Saudi categories remained minimal. The residence data showed a diverse spread, with the central region having a higher mild depression (12.7%). The duration of epilepsy presented a relatively uniform distribution across categories, with mild depression being slightly more prevalent in the "less than two years" group (14.6%). Analysis by type of epilepsy indicated that generalized epilepsy had the highest mild depression rate (15.9%). The P-values ranging from 0.406 to 0.684 imply a lack of statistical significance in these observations. 

**Table 3:** Prevalence of depression and its relationship with sociodemographic characteristics of the patients

Depression		Mild	Minimal	Moderate	Moderately severe	Severe	Total	P-value
Age	16-24	15(9.6)	6(3.8)	8(5.1)	3(1.9)	1(0.6)	33(21.0)	0.599
	25-34	20(12.7)	13(8.3)	16(10.2)	15(9.6)	2(1.3)	66(42.0)	
	35-44	10(6.4)	3(1.9)	11(7.0)	5(3.2)	2(1.3)	31(19.7)	
	45≤	9(5.7)	2(1.3)	11(7.0)	4(2.5)	1(0.6)	27(17.2)	
Gender	Female	23(14.6)	8(5.1)	25(15.9)	11(7.0)	3(1.9)	70(44.6)	0.505
	Male	31(19.7)	16(10.2)	21(13.4)	16(10.2)	3(1.9)	87(55.4)	
Nationality	Saudi	50(31.8)	24(15.3)	44(28.0)	24(15.3)	6(3.8)	148(94.3)	0.446
	Non-Saudi	4(2.5)	0(0.0)	2(1.3)	3(1.9)	0(0.0)	9(5.7)	
Residence	South	5(3.2)	2(1.3)	11(7.0)	2(1.3)	1(0.6)	21(13.4)	0.406
	East	11(7.0)	3(1.9)	5(3.2)	7(4.5)	0(0.0)	26(16.6)	
	North	9(5.7)	2(1.3)	4(2.5)	3(1.9)	0(0.0)	18(11.5)	
	West	9(5.7)	6(3.8)	12(7.6)	5(3.2)	3(1.9)	35(22.3)	
	Central	20(12.7)	11(7.8)	14(8.9)	10(6.4)	2(1.3)	57(36.3)	
Duration of epilepsy	Less than 2 years	23(14.6)	10(6.4)	16(10.2)	8(5.1)	2(1.3)	59(37.6)	0.684
	2-5 Years	12(7.6)	4(2.5)	9(5.7)	8(5.1)	3(1.9)	36(22.9)	
	More than 5 years	19(12.1)	10(6.4)	21(13.4)	11(7.0)	1(0.6)	62(39.5)	
Type	Focal	9(5.7)	4(2.5)	8(5.1)	7(4.5)	3(1.9)	31(19.7)	0.563
	Don’t know	20(12.7)	11(7.0)	22(14.0)	11(7.0)	1(0.6)	65(41.4)	
	Generalized	25(15.9)	9(5.7)	16(10.2)	9(5.7)	2(1.3)	61(38.9)	

The study further evaluates depression levels in relation to employment type, number of medications, and a history of mental illness; results are shown in Table 4. For mild depression, the highest prevalence was observed among the employed group (19.1%), followed by those on polytherapy (17.2%) and those with no mental illness (16.6%). However, the moderately severe category showed a spike among the unemployed (8.3%) and those with a history of mental illness (10.2%). The minimal depression group saw the highest numbers in employed individuals (11.5%) and those without a history of mental illness (10.2%). Moderate depression was most prevalent among employed participants (16.6%) and was significantly high in those with a history of mental illness (21.0%). Individuals on polytherapy showed an elevated rate of 17.8% in this category. Finally, severe depression, though relatively low in prevalence, was found to be slightly higher in those with a history of mental illness (2.5%). Notably, students and those on monotherapy had minimal representation in the severe depression category. This multifaceted analysis provides a comprehensive view of how employment status, medication type, and past mental health can intersect with depression severities.

**Table 4:** Comparison of depression with employment, drug therapy, number of drugs, and mental history Student vs. unemployed p=0.4911, student vs. employed p=0.06, employed vs. non employed p=0.11 No therapy vs. polytherapy p<0.02, no therapy vs. monotherapy p<0.008, polytherapy vs. monotherapy p<0.05 Mental Illness p=0.405 Employment type P-value: student vs. unemployed p=0.4911, student vs. employed p=0.06, employed vs. non employed p=0.11 Medication type P-value: no therapy vs. polytherapy p<0.02, no therapy vs. monotherapy p<0.008, polytherapy vs. monotherapy p<0.05 History of illness P-value: mental illness p=0.405

	Employment type			Number of medications			History of mental illness	
	Student	Unemployed	Employed	None	Polytherapy	Monotherapy	No	Yes
Mild	14 (8.9)	10(6.4)	30(19.1)	3(1.90)	27(17.2)	24(15.30)	26(16.6)	28(17.8)
Minimal	3(1.9)	3(1.9)	18(11.5)	2(1.30)	10(6.4)	12(7.60)	16(10.2)	8(5.1)
Moderate	9(5.7)	11(7.0)	26(16.6)	1(0.60)	28(17.8)	17(10.80)	13(8.3)	33(21.0)
Moderately severe	4(2.5)	13(8.3)	10(6.4)	2(1.30)	11(7.00)	14(8.90)	11(7.0)	16(10.2)
Severe	0(0.0)	3(1.9)	3(1.9)	2(1.30)	3(1.90)	1(0.60)	2(1.3)	4(2.5)

## Discussion

This is a cross-sectional study that aimed to assess the prevalence of depression among epileptic patients in Saudi Arabia. We also explored some of the sociodemographic as well as clinical variables and their relation with the occurrence of depression among patients with epilepsy. Our self-administered questionnaire was constructed based on previous study questionnaires where a validated Arabic-translated version of the patient health questionnaire PHQ-9 depression score was used [10].

The assessment of depression scores using PHQ9 revealed that the majority (87%) of the patients exhibited signs of depression, and a severe form of depression was identified in 3.8% of the patients while a moderately severe form was observed in 17.2%. A similar pattern was observed in Mubarak AA et al. study, in Taif city, Saudi Arabia [10]. Studies across different regions in the world demonstrated a high prevalence of depression among people with epilepsy, the possible explanation however is controversial [12]. The psychosocial factors associated with epilepsy like unemployment, stigmatization, and social isolation could be contributing to the occurrence of depression [12]. Anti-epileptic medications such as carbamazepine, gabapentin, lacosamide, lamotrigine, pregabalin, and valproate have psychiatric implications [12]. Neurobiological factors can also play a role in the pathogenesis of depression in epilepsy. Mazarati AM et al. study found an association between hippocampal IL-1β and epilepsy-associated depression [13].

When comparing the prevalence of depression in relation to age, we found increasing prevalence among those between 16 to 34 years of age with a decline in ages 35 and older. This was in line with Mubarak AA et al. study, in Taif City, Saudi Arabia but contradicted Thomson et al. study, possibly because of the major differences in study design and sample size [10,14]. Also, when it comes to gender difference, female predominance was reported, and this may be because of the sample size difference and sampling methods [6]. Higher rates of depression were observed in those who had the illness for more than five years. Similarly, several studies reported more severe depression is associated with longer duration of epilepsy [10,14-16]. This is possible because of the higher social and financial burden associated with chronic diseases. In previous studies, depression was observed more in patients with a generalized type of seizure, which was also seen in our study [10]. Generalized seizures involve loss of consciousness and postictal symptoms that can affect the patient's daily functioning [12].

Caetano et al. study showed that depression was commonly seen in unemployed epileptics [17]. In our study, employed epileptics showed a spike in mild depression, while unemployed ones showed a spike in moderate depression. The financial burden associated with unemployment could be a contributing factor. Regarding the number of medications, higher rates of depression were most prevalent among those on polytherapy, and this finding was statistically significant (p<0.05 at 95%CI). Tegegne et al. study found that patients on two or more medications were twice as likely to have depression [5]. This is expected since those who are on multiple anti-epileptic medications tend to have more refractory seizures. A study found that patients with uncontrolled seizures have double the prevalence of major depression compared with patients with controlled seizures [18].

Limitations

The robustness of our research findings may be compromised due to the limited sample size. Furthermore, the disproportionate gender distribution, with a preponderance of female participants, introduces potential biases. Additionally, the utilization of a self-administered electronic questionnaire and identifying those who have epilepsy by asking them might not be the most reliable for outcome measurement as patients might be hesitant to speak about their illness or address it. 

## Conclusions

In conclusion, our study aimed to assess the prevalence of depression among epileptic patients in Saudi Arabia. We also examined the relationship between various sociodemographic and clinical variables associated with depression in epilepsy. We found that the majority of epileptic patients have depression symptoms. We also found a statistically significant association between the number of medications and depression in epilepsy. More research utilizing different methodologies and a larger sample size needs to be conducted to obtain more representative results that could improve the outcomes in patients with epilepsy and co-morbid depression.
